# Management of the Infrapatellar Fat Pad in Total Knee Arthroplasty: A Comprehensive Review of Recent Evidence

**DOI:** 10.7759/cureus.99350

**Published:** 2025-12-16

**Authors:** Ege Islatince, Sridhar R Sampalli

**Affiliations:** 1 Trauma and Orthopaedics, Salisbury District Hospital, Salisbury, GBR

**Keywords:** anterior knee pain, fat pad excision, fat pad preservation, functional outcomes, hoffa's fat pad, infrapatellar fat pad, knee surgery, patellar tendon length, rehabilitation, total knee arthroplasty

## Abstract

Surgical management of the infrapatellar fat pad (IPFP) during total knee arthroplasty remains debated: excision may improve exposure but risks tendon changes and pain, while preservation protects soft tissues yet limits visualisation. This review synthesises randomised trials and meta-analyses (2020-2025) comparing IPFP excision versus preservation. Outcomes, including anterior knee pain, functional scores such as Knee Society Score (KSS) and the Knee Injury and Osteoarthritis Outcome Score-Quality of Life subscale (KOOS-QoL), range of motion (ROM), patellar tendon length, and Insall-Salvati ratio (ISR), were evaluated at less than or equal to three months and 6-12 months. Evidence shows modest tendon shortening after excision without lasting differences in pain, function, or mobility. Preservation may offer small early recovery benefits, but long-term outcomes are equivalent. Intraoperative decisions should balance exposure needs with potential early advantages of preservation.

## Introduction and background

Knee osteoarthritis (OA) is among the most prevalent musculoskeletal conditions globally, affecting over 374 million people as of 2021 and accounting for approximately 4.3% of the world’s population [1]. The global, United Kingdom (UK)-specific, and age/sex-specific prevalence rates, as well as principal risk factors, are summarised in Table 1 [1-3]. Prevalence continues to rise worldwide, driven by ageing populations, urbanisation, increasing obesity rates, and changing patterns of physical activity, with similar trends now observed in low- and middle-income countries [1,4]. In the United States, more than 14 million adults are affected, while over five million individuals in the UK experience symptomatic disease [1,2]. OA is more common in women, with incidence peaking between ages 55 and 64 [3-5]. Projections suggest that the global prevalence of knee OA will increase by nearly 75% by 2050 [2], contributing to substantial disability, reduced quality of life (QoL), and rising healthcare and productivity costs [2,4,6].

**Table 1:** Summary of Prevalence, Morbidity, and Risk Factors for Knee Osteoarthritis Table synthesised from global reports, UK national statistics, and recent peer-reviewed publications. Prevalence rates, disability-adjusted life years (DALYs), and risk factor information are compiled from sources including the Global Burden of Disease studies, Versus Arthritis statistics, the National Institute for Health and Care Excellence (NICE), major cohort studies, and systematic reviews published between 2023 and 2025.

Category	Data/Statistic	Reference/Notes
Global Prevalence (2021)	374.7 million people affected worldwide (~4.3% prevalence rate)	Versus Arthritis [2]
UK Prevalence	5.4 million people with knee osteoarthritis (OA)	Global Burden of Disease Study [1]
Sex Distribution	Higher prevalence in women (1.7× higher than men)	Noble et al. [3]
Age Distribution	Most common in adults aged 50+; peak prevalence ages 55-64	NICE [5]
Projected Global Trend	Cases expected to increase by 74.9% for knee OA by 2050	Versus Arthritis [2]
Morbidity	10-20% of patients are not fully satisfied after treatment; 31-54% report persistent pain or functional problems	Noble et al. [3]
Major Risk Factors	Older age, female sex, obesity/high BMI, previous knee injury, genetics, metabolic syndrome, joint stress	Geng et al. [4]

Total knee arthroplasty (TKA) is the definitive treatment for end-stage OA, with more than 3.6 million procedures performed globally in 2023 [5-7]. In the United States, annual primary TKA volume exceeded 715,000 inpatient procedures in 2018, with projections estimating more than 1.26 million cases by 2030 [5-7]. The UK maintains one of the largest arthroplasty registries internationally, reporting more than 100,000 TKAs annually [6,7]. Despite these favourable outcomes, up to 20% of patients continue to experience persistent postoperative pain, stiffness, or functional limitations after TKA [6]. These findings highlight the need to identify perioperative predictors of poor postoperative recovery [6]. One potentially modifiable but insufficiently explored factor is infrapatellar fat pad (IPFP) fibrosis [8].

The IPFP is an intracapsular but extrasynovial adipose structure that contributes to biomechanical cushioning, immune regulation, and nociceptive signalling in the knee joint [9,10]. Its dense innervation and peripheral vascularity make it susceptible to inflammation and fibrotic remodelling [9-11]. The IPFP secretes pro-inflammatory cytokines and adipokines - such as interleukin-1β (IL-1β), tumour necrosis factor-α (TNF-α), and nerve growth factor (NGF) - that can amplify synovial inflammation and pain sensitisation in OA and postoperative states [10-12]. These biological properties have led to growing interest in the IPFP as a potential contributor to postoperative pain patterns.

Historically, the IPFP was routinely resected during TKA to improve visualisation of the operative field, with excision rates exceeding 80% in earlier decades [13]. Concerns regarding patellar tendon shortening, loss of biomechanical cushioning, and increased anterior knee pain subsequently shifted interest toward preservation strategies [14,15]. Recent randomised trials and meta-analyses demonstrate that although excision results in modest shortening of the patellar tendon, it does not lead to significant long-term differences in pain, function, or mechanical outcomes compared with preservation [14-16]. Preservation may offer short-term reductions in subacute anterior knee pain, though these benefits typically resolve within three months [15].

Major clinical guidelines - including those from the American Academy of Orthopaedic Surgeons (AAOS), the National Institute for Health and Care Excellence (NICE), and the European Society of Sports Traumatology, Knee Surgery and Arthroscopy (ESSKA) - recommend individualised intraoperative decision-making to balance surgical exposure with soft tissue preservation [17,18]. Evidence supporting these recommendations includes a randomised controlled trial (RCT)-only meta-analysis published in 2020, which found no long-term functional disadvantages associated with IPFP excision [14]. A double-blind randomised clinical trial from 2024 similarly demonstrated that early postoperative differences between excision and preservation resolved by three months [15]. Additional RCTs comparing anterior knee pain, functional scores, range of motion (ROM), and patellar tendon metrics consistently report clinical equivalence between the two approaches [14-13]. Multiple systematic reviews further support that neither excision nor preservation confers superior long-term outcomes after TKA [13,19].

## Review

Methods

This study is a structured review of published RCTs and meta-analyses, rather than a retrospective database analysis. A comprehensive search was conducted in PubMed, Web of Science, Embase, and the Cochrane Library using a predefined combination of Medical Subject Headings (MeSH) terms and free-text keywords relevant to IPFP pathology and total knee arthroplasty (TKA). The MeSH strategy included “Infrapatellar Fat Pad”[MeSH], “Adipose Tissue”[MeSH], “Arthroplasty, Replacement, Knee”[MeSH], “Knee Joint”[MeSH], “Pain Measurement”[MeSH], and “Postoperative Pain”[MeSH], combined with keywords such as “IPFP,” “fat pad,” “excision,” “resection,” “preservation,” “fibrosis,” and “anterior knee pain.” Boolean operators included (“Infrapatellar Fat Pad” OR “IPFP” OR “fat pad”) AND (“Arthroplasty, Replacement, Knee” OR “TKA”) AND (“excision” OR “resection” OR “preservation”).

The initial search covered studies published between January 2020 and 31 May 2025. In response to the reviewer’s request for a new updated search run, an additional search covering June 2025 to the present was conducted using the same MeSH-based strategy. This updated search did not identify any new RCTs or meta-analyses directly comparing IPFP excision versus preservation during primary TKA. Post-2025 publications were predominantly observational, biomechanical, or imaging-based investigations and therefore did not meet the predefined eligibility criteria. As a result, no new evidence was found that changed or supplemented the conclusions of the original review.

Eligibility Criteria

Inclusion criteria were (i) publication date between January 2020 and 31 May 2025; (ii) study design restricted to RCTs or meta-analyses; (iii) involved adult patients undergoing primary TKA; (iii) direct comparison of IPFP excision versus preservation during primary TKA; and (iv) reported outcomes including pain, ROM, Knee Society Scores (KSS), tendon morphology, or QoL.

Exclusion criteria were (a) cadaveric or animal studies; (b) study protocols without published results; (c) non-English publications; and (d) studies involving pediatric populations or revision TKA, or that did not include a direct comparison between IPFP excision and preservation.

Rationale for Excluding Systematic Reviews

Systematic reviews were not included because the aim of this study was to synthesise primary randomised evidence. Systematic reviews may pool heterogeneous study designs, often include non-randomised studies, and do not contribute new primary outcome data. Therefore, they were unsuitable for addressing the specific comparative clinical endpoints defined in our protocol.

Study Selection and Review Process

**Figure 1:**
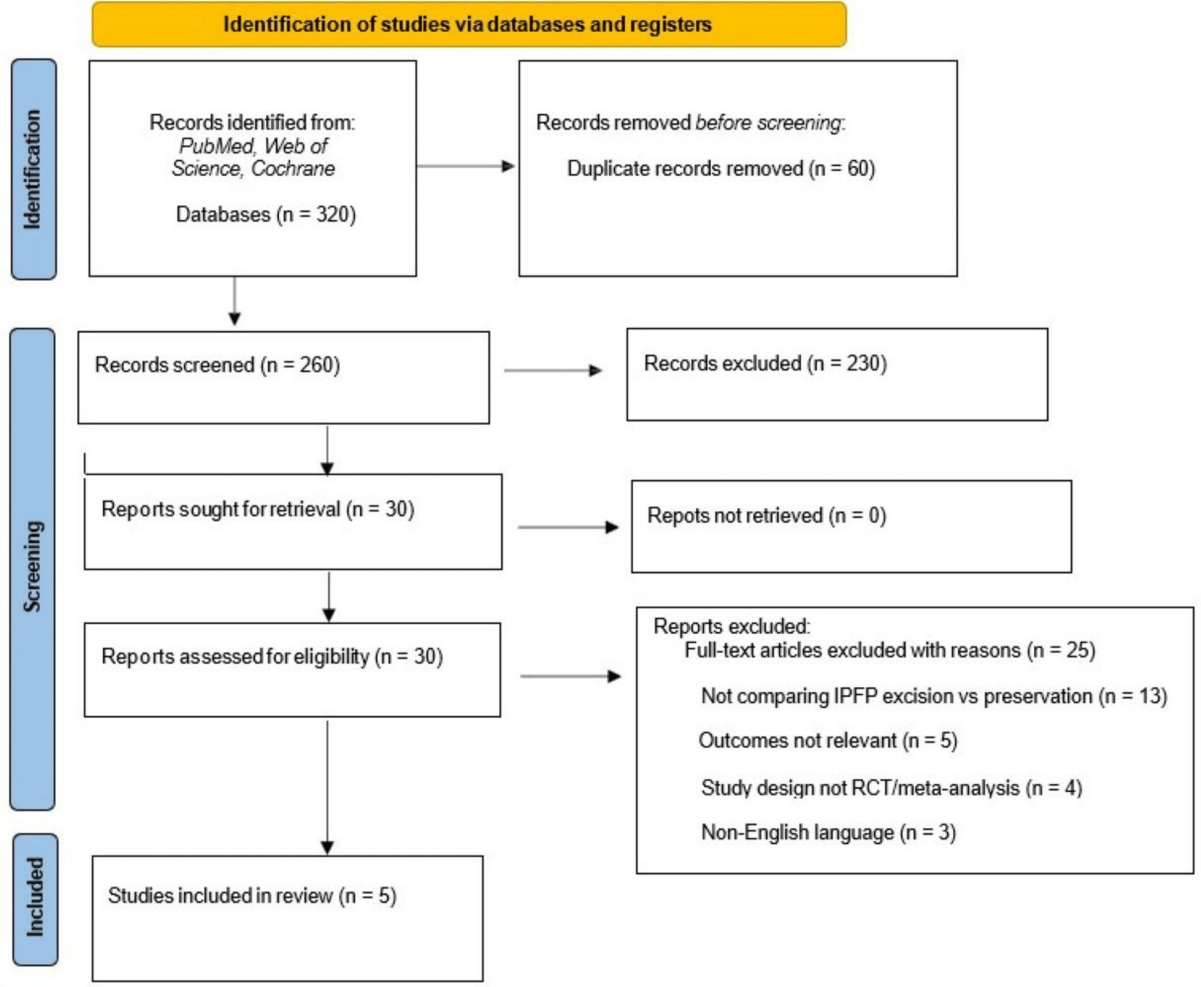
PRISMA 2020 Flow Diagram for Study Selection Process PRISMA: Preferred Reporting Items for Systematic Reviews and Meta-Analyses; IPFP: infrapatellar fat pad; RCT: randomised controlled trial

Study selection involved screening titles and abstracts, followed by full-text eligibility assessment. Two reviewers independently performed study selection, data extraction, and risk-of-bias evaluation, with disagreements resolved by consensus. The search and selection processes adhered to the Preferred Reporting Items for Systematic Reviews and Meta-Analyses (PRISMA) 2020 guidelines [20]. The screening and inclusion workflow is presented in Figure 1. No geographic restrictions were applied, and included RCTs originated from multiple countries according to where each primary study was conducted.

Results

The primary outcomes evaluated in this review were selected for their clinical relevance and widespread validation in orthopaedic research [21,22]. Pain outcomes, including early postoperative and chronic anterior knee pain, are essential for assessing patient satisfaction and detecting IPFP-related complications [23]. Functional measures such as the KSS and the Knee Injury and Osteoarthritis Outcome Score-Quality of Life subscale (KOOS-QoL) are recommended indicators of knee function, mobility, and overall joint health [3,22]. Objective parameters, including ROM, patellar tendon length (PTL), and the Insall-Salvati ratio (ISR), allow evaluation of anatomic and mechanical effects following TKA [24]. A 2020 meta-analysis of nine RCTs (783 TKAs) demonstrated that IPFP excision resulted in approximately 4.5 mm of PTL shortening at 6-12 months, without differences in ISR, anterior knee pain, KSS, or ROM compared with preservation [14]. A double-blind RCT by Benner et al. reported lower KOOS-QoL scores in the excision group at six weeks, though these differences resolved by three months [25]. That same trial found a modest early advantage in knee extension favouring excision at three months, but no differences between groups in KOOS subscales, KSS, ROM, PTL, or ISR by 12 months [25].

Rates of achieving Minimal Clinically Important Difference (MCID) and Patient Acceptable Symptom State (PASS) for pain and function did not differ between excision and preservation groups at three or 12 months [15]. A 2022 RCT comparing 120 primary TKAs found no significant differences between groups in anterior knee pain, KSS, or patellar tendon measurements during follow-up to 12 months [16]. A 2019 systematic review of eight comparative studies similarly reported no differences in chronic anterior knee pain, functional outcomes, or ROM between groups up to 18 months [13]. A 2021 systematic review pooling 1,132 TKAs confirmed that resection and preservation yield equivalent postoperative outcomes for pain, KSS, ROM, and patellar tendon parameters through two years [19]. The main characteristics of included trials and meta-analyses are summarised once in Table 2 to avoid duplication.

**Table 2:** Characteristics of Included Studies IPFP: infrapatellar fat pad; TKA: total knee arthroplasty; RCT: randomised controlled trial; PTL: patellar tendon length; ISR: Insall-Salvati ratio; KSS: Knee Society Score; ROM: range of motion; KOOS-QoL: Knee Injury and Osteoarthritis Outcome Score-Quality of Life; VAS: visual analogue scale; WOMAC: Western Ontario and McMaster Universities Osteoarthritis Index; RR: risk ratio; MD: mean difference

Author, Year	Study Design	Sample Size	Intervention	Primary Outcomes	Follow-Up	Key Findings
Sun et al., 2020 [14]	Meta-analysis of RCTs	9 RCTs, 783 TKAs	IPFP excision vs. preservation	PTL, ISR, anterior knee pain, KSS, ROM	6-12 months	PTL shortening ~4.5mm with excision (p<0.05); no differences in ISR, pain, KSS, ROM
Benner et al., 2024 [15]	Double-blind RCT	100 patients	IPFP excision vs. preservation	KOOS-QoL, KSS, ROM, PTL, ISR	6 weeks, 3 months, 12 months	KOOS-QoL 10 points lower at 6 weeks with excision (p=0.03); resolved by 3 months; no differences at 12 months
							Fahmy and Seifeldin, 2022 [16]	RCT	120 patients (60 per arm)	IPFP excision vs. preservation	Anterior knee pain (VAS), KSS, PTL, ISR	3, 6, 12 months	No significant differences in pain (p=0.42), KSS function (p=0.36), PTL, or ISR at any time point
Nisar et al., 2019 [13]	Systematic review	8 studies, 894 TKAs	IPFP excision vs. preservation	Chronic anterior knee pain, Oxford Knee Score, KSS, ROM	Up to 18 months	Similar chronic pain rates (14% vs. 16%, p=0.48); slight early WOMAC function advantage for preservation at 3 months (p=0.04)
Yao et al., 2021 [19]	Systematic review	11 studies, 1,132 TKAs	IPFP resection vs. preservation	Anterior knee pain, KSS, ROM, PTL, ISR	6-24 months	No significant differences: pain RR 1.04 (0.89-1.21), KSS MD -0.8 (-3.2 to 1.6), ROM MD -1.5 (-3.8 to 0.8)

Discussion

Insights from Hoffa’s disease and fat pad impingement in non-arthroplasty populations provide relevant contextual parallels for TKA outcomes [25]. In Hoffa’s syndrome, fibrotic scarring and oedema within the IPFP can produce chronic anterior knee pain that is often refractory to rehabilitation and may require arthroscopic debridement [25]. Although TKA cohorts differ due to periarticular tissue disruption, these parallels suggest that extensive fibrosis may predispose some patients to amplified postoperative discomfort [25]. RCTs in TKA populations demonstrate that selective IPFP excision can mitigate fibrotic pain sources without impairing patellar tracking or extensor mechanism strength [25]. Imaging studies in OA patients further show that MRI-graded IPFP fibrosis correlates with pain severity and synovitis scores, reinforcing the concept of fibrosis as a modifiable therapeutic target [25]. Applying these mechanistic insights to TKA practice may allow preoperative MRI-based stratification to identify patients most likely to benefit from IPFP resection [25]. Future research should evaluate whether preoperative MRI-based stratification, particularly fibrosis grading and signal intensity patterns, can reliably identify patient subgroups who may derive greater benefit from selective IPFP resection versus preservation.

Clinical Rationale and Implications

Adequate visualisation of the femoral and tibial articular surfaces is essential for achieving accurate component alignment and cementation during TKA [14]. Historically, routine IPFP excision was recommended to improve surgical exposure, especially in cases of limited flexion or tight deformities [13]. The IPFP, however, is now recognised as a biomechanically active structure that contributes cushioning, immune signalling, and nociceptive modulation through its rich vascular and neural networks [9]. In our review, IPFP excision was associated with a mean patellar tendon shortening of approximately 4.5 mm at 6-12 months, without clinically meaningful changes in the ISR [14]. Early postoperative increases in anterior knee pain and lower KOOS-QoL scores observed after excision resolved by the three-month follow-up [25]. No significant differences in pain, functional outcomes, or tendon parameters were present between groups by 12 months [25]. Patient-specific factors such as body mass index (BMI), preoperative ROM, and history of prior knee surgery may help guide individualised IPFP management [3]. Obese patients tend to have thicker, more fibrotic fat pads and reduced flexion, making resection potentially more beneficial for achieving adequate exposure [10]. By contrast, non-obese patients with good baseline mobility may benefit from preservation to maintain soft-tissue cushioning and reduce early postoperative discomfort [23]. Prior knee surgeries, particularly arthroscopy involving fat-pad debridement, can increase scarring and alter tissue planes, suggesting that surgeons may need to adapt resection extent based on intraoperative findings [15].

Mechanistic Insights From Pathophysiology Studies

Mechanistic investigations provide important insights into the biological role of the IPFP in OA and postoperative recovery [26]. Histological studies have demonstrated that IPFP fibrosis is associated with elevated levels of proinflammatory cytokines such as IL-1β and TNF-α, as well as increased NGF expression contributing to nociceptive sensitisation and synovitis [26]. Animal models show that partial resection of inflamed IPFP reduces local cytokine concentrations and attenuates early postoperative pain, supporting its role as a potentially modifiable pain generator [26]. Conversely, IPFP preservation maintains biomechanical shock absorption and protects the retropatellar vascular plexus, which may help reduce early postoperative tissue trauma and effusion formation [26]. Translational research suggests that molecular markers, such as synovial fluid NGF levels, may help identify patients who could derive greater benefit from IPFP resection versus preservation [26]. The IPFP also functions as an active endocrine organ by secreting adipokines, including leptin and adiponectin, which influence chondrocyte apoptosis and matrix degradation [26]. Recent single-cell transcriptomic studies have identified proinflammatory macrophage subsets within the IPFP that amplify cytokine cascades in osteoarthritic joints [26]. Understanding these cellular populations may enable the development of targeted therapies, such as local anti-NGF injections, to reduce fibrosis and pain without requiring surgical excision [26].

Comparisons With Non-arthroplasty IPFP Conditions

Insights from Hoffa’s disease and fat pad impingement in non-arthroplasty populations provide relevant contextual parallels for TKA outcomes [25]. In Hoffa’s syndrome, fibrotic scarring and oedema within the IPFP can produce chronic anterior knee pain that is often refractory to rehabilitation and may require arthroscopic debridement [25]. Although TKA cohorts differ due to periarticular tissue disruption, these parallels suggest that extensive fibrosis may predispose some patients to amplified postoperative discomfort [25]. RCTs in TKA populations demonstrate that selective IPFP excision can mitigate fibrotic pain sources without impairing patellar tracking or extensor mechanism strength [25]. Imaging studies in OA patients further show that MRI-graded IPFP fibrosis correlates with pain severity and synovitis scores, reinforcing the concept of fibrosis as a modifiable therapeutic target [25]. Applying these mechanistic insights to TKA practice may allow preoperative MRI-based stratification to identify patients most likely to benefit from IPFP resection [25].

Limitations and Methodological Considerations

Several limitations should be considered when interpreting these findings. Most RCTs included relatively small samples (60-120 patients) and were powered primarily for early postoperative pain outcomes rather than infrequent mechanical complications such as patella baja or tendon rupture. Follow-up duration in the available trials rarely exceeded 12 months, limiting evaluation of late sequelae, including accelerated patellar wear or revision risk. Surgical heterogeneity, including variability in resection depth, instrumentation, tourniquet application, and cementation technique, introduces confounding that restricts definitive pooling of effect sizes across studies. Outcome measures were not entirely uniform; although standardised functional scores such as KSS and KOOS-QoL were frequently used, thresholds for MCID were inconsistently applied. Additionally, few trials systematically graded preoperative IPFP fibrosis or quantified intraoperative exposure benefits, limiting patient-specific stratification and refined surgical decision-making. Important operative variables such as tourniquet duration, cement viscosity, and patellar resurfacing status were also insufficiently reported, despite their potential influence on tendon morphology and postoperative pain. Beyond study-level issues, this review is limited by its narrative design rather than a systematic review approach, which may increase susceptibility to selection bias despite adherence to predefined eligibility criteria. Finally, only English-language studies were included, which may exclude relevant evidence published in other languages and thereby narrow the generalizability of conclusions.

## Conclusions

Taken together, these data support two central conclusions. First, IPFP excision causes a small anatomical change without lasting functional penalty as patients recover equivalent pain relief, mobility, and QoL by three months and sustain these outcomes through at least one year. Second, IPFP preservation may confer minor early benefits - slightly less subacute pain or functional limitation - but these advantages are transient and do not influence long-term outcomes. Thus, IPFP management during TKA should be individualised: preserve the fat pad when adequate exposure can be achieved, and early subacute comfort is prioritised; excise when visualisation is otherwise compromised, knowing that long-term tendon integrity and patient satisfaction will not be compromised. Limitations of the current evidence include heterogeneity in outcome definitions, modest sample sizes for some endpoints, and limited follow-up beyond two years. Standardisation of imaging protocols for tendon metrics and consistent use of validated patient‐reported outcome measures in future trials would enhance comparability. Investigation of IPFP fibrosis pathways via imaging and histology may clarify mechanistic links to post‐TKA pain, but should be framed as exploratory until supported by randomised data. To foster standardised, evidence-based IPFP protocols, multicenter collaborations should establish consensus on fibrosis grading, surgical technique, and long-term outcome monitoring. Only through coordinated research efforts can we fully elucidate the optimal management of the IPFP in diverse TKA populations.
